# The Effect of Meteorological, Pollution, and Geographic Exposures on Death by Suicide: A Scoping Review

**DOI:** 10.3390/ijerph18157809

**Published:** 2021-07-23

**Authors:** Sarah L. Cornelius, Tara Berry, Amanda J. Goodrich, Brian Shiner, Natalie B. Riblet

**Affiliations:** 1 VA Medical Center, White River Junction, VT 05009, USA; tara.berry@va.gov (T.B.); brian.shiner@va.gov (B.S.); natalie.riblet@va.gov (N.B.R.); 2 Department of Population and Public Health Sciences, University of Southern California, Los Angeles, CA 90033, USA; ajgoodri@usc.edu; 3 Geisel School of Medicine, Dartmouth College, Hanover, NH 03755, USA

**Keywords:** suicide, meteorology, weather, pollution, geography, altitude

## Abstract

Suicide is a significant public health concern worldwide and in the United States. Despite the far-reaching impact of suicide, risk factors are still not well understood and efforts to accurately assess risk have fallen short. Current research has highlighted how potentially modifiable environmental exposures (i.e., meteorological, pollution, and geographic exposures) can affect suicide risk. A scoping review was conducted to evaluate the strength of the historical and current literature on the environment’s effect on suicide and suicide risk. Three databases (i.e., Medline, Embase, and PsychInfo) were reviewed to identify relevant studies and two authors independently reviewed studies considering pre-determined inclusion criteria. A total of 46 meteorological studies were included as well as 23 pollution studies and 12 geographic studies. Descriptive statistics, including counts, percentages, review of studies’ sample size (minimum, maximum, median, and interquartile range), were calculated using Excel and SAS 9.4. Overall, strong evidence supports that exposure to sunlight, temperature, air pollution, pesticides, and high altitude increases suicide risk, although effect sizes range from very small to small.

## 1. Introduction

Suicide is a public health concern globally. In fact, death by suicide accounted for 1.4% of all deaths worldwide in 2016, making it the 18th leading cause of death in the world [1]. It is also a substantial concern in the United States, where suicide accounted for more than 47,500 deaths in 2019, making it the 10th leading cause of death [2]. Furthermore, many more people experience suicidal thoughts or make a suicide attempt; according to the Centers for Disease Control and Prevention, 12 million American adults seriously thought about suicide as an option in 2018 [2]. Despite the far-reaching impact of suicide, risk factors are still not well understood. Current research has identified several factors involved in suicide risk assessment, including individual-level factors (e.g., history of depression or other mental illness, history of suicide attempts, demographic characteristics), community- or sociological-level factors (e.g., inadequate community connectedness or health policy), and biological factors (e.g., genetic, epigenetic, neurobiological, or metabolic) [3,4,5,6,7]. These factors are mirrored among the most high-risk individuals; clinical, psychological, sociological, and neurobiological or biological risk factors may play the largest role in suicidal behavior [8]. Furthermore, in addition to the neurobiological basis for suicide, nuanced psychological topics, such as hopelessness, also emerge in individuals at highest risk of suicide [9,10]. This suggests that both biological and non-biological factors can and do intersect. Suicide is a complex combination of factors, and truly warrants this emerging field of research. Historically, suicide researchers have also observed suicide rates following a cyclical or seasonal pattern, with rates peaking in spring and early summer [11,12], which challenges risk assessments involving only psychological, sociological, or biological variables [13]. This seasonality observation has been replicated throughout the world, though a reciprocal pattern is observed between the Northern and Southern hemispheres corresponding to spring and summer months in each respective location [13,14]. Other environmental factors (e.g., meteorology, pollution, or geography) are less well understood, but may either mediate the effect of seasonality or independently impact suicide risk [14]. As a result, these modifiable or potentially treatable risk factors are significant considerations for research.

A review by Woo, Okusaga and Postolache (2012) highlighted literature supporting the role of bioclimatic factors, global geography, pollution, and season as possible risk factors for suicide [14]. Meteorological factors included in their review were temperature, sunlight, and precipitation. Studies reported that increased intensity or exposure to sunlight was associated with increased risk of suicide [14]. Similarly, high temperatures resulted in a modest increase in suicide rates in the two studies included in their review. Precipitation may affect suicide risk, but the studies included in their review did not report significant associations [14]. They also reviewed air quality variables such as air pollution and allergens, reporting that increased levels of pollutants are correlated or associated with increased suicide rates and risk. These observations are further supported by other standardized reviews, which considered several pollutants, including particulate matter of less than 2.5 micrometers (PM_2.5_) and less than 10 micrometers (PM_10_), nitrogen dioxide (NO_2_), ozone (O_3_), and sulfur dioxide (SO_2_) [15,16]. Geography variables, such as altitude of residence, may also play a significant role in suicide risk due to causing chronic mild hypoxia [17]. Reviews and observational studies have demonstrated increasing suicide rates or risk with increasing altitude of residence [17]. This review will focus on the strength of association between suicide and three main categories of potentially modifiable environmental factors: meteorology, pollution, and geography. As established, these factors have shown some evidence to be associated with suicide, but studies have reported contradictory results [14]. Subsequently, a review considering the strengths of these studies will aid in the understanding of how the environment affects suicide risk.

To date, there has not been a comprehensive scoping review assessing the role of meteorological, pollution, and geographic environmental factors on suicide outcomes. With the growing concern about the rise in suicide in the population and the possible modifiable role of the environment on public health outcomes, a thorough review of the current literature on how the environment may affect suicide is warranted. The aim of this scoping review is to broadly review current advances in this field, with a focus on three of the most well-researched categories (i.e., meteorology, pollution, and geography) and their relationship to death by suicide. Specifically, we aim to analyze the strength of the evidence available on each of these factors with regard to death by suicide. The flexibility of the scoping review allowed us to include a wider range of studies and analyses on the topic, and thus provide a more thorough review of where the research currently stands.

## 2. Materials and Methods

### 2.1. Study Search and Inclusion

We searched Medline, Embase, and PsychInfo to identify studies that addressed our research topic from inception through 1 September 2020. Within each database, we ran a search strategy to identify studies that addressed one or more of the three topic areas and their intersection with death by suicide. The approach to the search in each database can be found in Table 1.

We initially identified MeSH terms and key words using Medline (PubMed). We used “OR” to include the environmental factor themes and “AND” to combine these themes to the suicide themes. Suicide themes included “Suicide” (MeSH), “Suicide, Attempted” (MeSH), “Suicide, Completed” (MeSH), “Suicide[ti]”, and Suicidal*[ti]”. The MeSH terms and key words were applied exactly to our Medline search and modified as necessary to accommodate the search structure of each of the remaining databases. For consistency across databases and to limit to studies with a focus on environmental factors and suicide, key words were searched specifically in the title only.

We included studies if they met four pre-defined criteria. First, the study focused on a meteorological, pollution, or geographic environmental exposure. Meteorological factors included the most commonly studied exposures: sunlight duration or intensity, temperature, humidity, air pressure, or precipitation. We did not include an analysis of dewpoint, cloud cover, typhoon signals, or windspeed as only one to two studies assessed each exposure [18,19,20,21]. Studies on seasonality alone were not included as the study team considered this to be a well-addressed field [13]. Pollution factors included air, land, water, light, or sound pollution. More specifically, exposures included particulate matter with a diameter 2.5 micrometers or less (PM_2.5_) or 10 micrometers or less (PM_10_), nitrogen dioxide (NO_2_), ozone (O_3_), and sulfur dioxide (SO_2_), all pesticides, toxic metals, and noise pollution. We did not include indoor pollution, such as smoking because these factors are often driven by individual behavior. In addition, we excluded pollutants that were evaluated as a suicide method, such as carbon monoxide poisoning, in the context of the study. We included environmental pollution variables associated with occupation, such as exposure to toxic substances (e.g., heavy metal or pesticides). Geographic factors included elevation or altitude. Second, we required that the study outcome was death by suicide as this is one of the most robust measures of suicide risk in a population [22]. Third, we required that the study design was an experimental, observational, or other type of quantitative or epidemiologic study. Reviews, editorials, and commentaries were not included in this review, but we did review these articles to locate any relevant studies that may have been missed during our primary search. Fourth, we required that all papers be written in or translated into English. Using the above approach, we identified a total of 613 potentially eligible studies. S.L.C. and T.B. then reviewed titles and abstracts of all studies considering our inclusion criteria using the Rayyan QCRI web application [23]. Disagreements and unclear assignments were discussed with N.B.R. in order to develop one comprehensive list.

### 2.2. Analysis

We began with a breakdown of study characteristics. We categorized each study by exposure(s), observation level (i.e., population vs. individual), and study results (i.e., significant vs. non-significant). Studies were included in each exposure group that they assessed, and thus a single study could contribute information on multiple exposures. A study was considered to have a significant association if the *p*-value associated with the overall population or at least one subpopulation was reported as *p* < 0.05. Row percentages were calculated in our initial breakdown of exposures. Numerators were the number of studies with a significant/non-significant result and denominators were the total number of studies in that exposure or observation group. This allows us to report the percentage of studies reporting a significant or non-significant result for each exposure and observation level.

We then calculated descriptive statistics to assess whether sample size differed among exposures, observation level, and significance. We reported sample size stratified by whether there was a statistically significant association between the exposure and death by suicide. Numerators were the number of studies reporting a sample size and denominators were the total number of studies in that exposure or observation group. This allowed us to determine whether there were differences in sample size reporting among certain groups (e.g., exposure groups or significance/non-significance groups). Among studies reporting a sample size, the number of suicides in the sample was compared using ranges; we reported the minimum and maximum sample sizes among each exposure group, observation level, and reported significance. Medians and interquartile ranges were then calculated to compare whether studies reporting significant results included a larger sample.

Among studies reporting a significant result, we reported the strength of association through reported effect sizes. Effect sizes were categorized according to Ferguson’s (2009) primer ranging from very small (i.e., “recommended minimum effect size”) to strong [24]. Row percentages were calculated as the number of studies reporting the specified effect size (e.g., “Very Small”) divided by the total number of studies reporting a significant result in each exposure group. We also reviewed the direction of association for each study. We considered a negative correlation coefficient or ratios less than 1.0 to be a negative association, and a positive correlation coefficient or ratios greater than 1.0 to be a positive association [25].

Lastly, we assessed study design through analysis of covariates. We stratified by each environmental factor (i.e., meteorological, pollution, and geography) and significance. We reported the number and percent of studies including measures of time and season. Measures of time included studies addressing the time between exposure and outcome (e.g., lag) while measures of season included adjustment, stratification, or matching by season or month variables. Confounders were categorized into environmental, individual-level factors, and community-level factors. Effect modification was categorized into the three most common categories: age, gender or sex, and suicide method (i.e., violent vs. non-violent methods). Percentages were calculated by dividing the number of studies including the covariate measure by the total number of significant or non-significant studies.

## 3. Results

**Figure 1:**
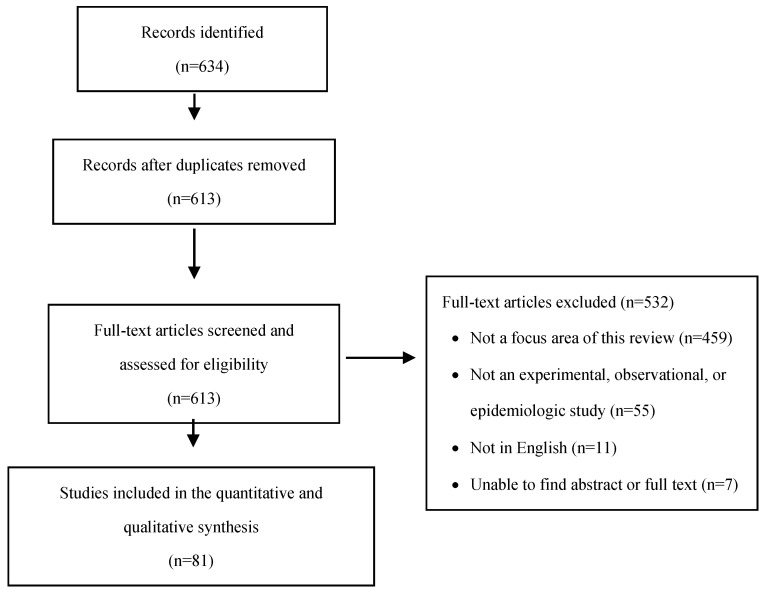
PRISMA flow diagram of study inclusion.

We identified 613 studies for this review, and subsequently included a total of 81 studies after assessing for eligibility (Figure 1). Of these 81 studies, 46 focused on meteorological factors, 23 on pollution factors, and 12 on geographic factors. References for each study included in this review are in Word S1.

### 3.1. Meteorological Factors

Among the 46 studies involving meteorological exposure variables, the majority of studies included population-level (73.9%) rather than individual-level (26.1%) outcomes. Over 80% of population-level (88.2%) and individual-level (83.3%) studies reported a significant association between a meteorological variable and death by suicide (Table 2). Exposure variables included sunlight, temperature, humidity, air pressure, and rainfall. Over half of the studies that evaluated sunlight intensity (60.0%), sunlight duration (65.2%), or temperature (78.9%) reported a significant association with death by suicide in at least one population included in the sample. The majority of studies assessing humidity (63.6%), air pressure (57.1%), and rainfall (81.3%) were more likely to report non-significant results.

Overall, sample size varied widely across all meteorological exposures, regardless of the reported results (Table 3). Among studies of temperature reporting a significant result, the median number of suicides was 22,564 compared to 5706 among studies reporting a non-significant result. Considering the former’s interquartile range (IQR) of nearly 85,000, the sample sizes among the two groups cannot be considered substantially different. Similar ranges were observed among the other meteorological exposures as well, suggesting that sample size did not differ greatly among studies reporting significant and non-significant results.

Effect sizes were more stable across studies. Among studies with significant results, the majority reported very small to small effect sizes and no studies reported a large effect size (Table 4). This was especially true for studies of sunlight intensity (very small: 66.7%; small: 0%), sunlight duration (very small: 40.0%); small: 40.0%), and temperature (very small: 66.7%; small: 26.7%). Notably, the direction of association was mixed among these studies. Two of the seven studies involving sunlight intensity reported a positive association with death by suicide while one study reported a negative association. Similarly, 12 studies assessing sunlight duration reported a positive association while three reported a negative association. Lack of power likely played a role in these discrepant results, particularly because the majority of studies of sunlight intensity and duration reported a very small to small effect size. Furthermore, sample size varied widely across studies, and thus some studies may not have had adequate power to detect such small effect sizes. Studies assessing temperature were more consistent. Twenty-nine studies reported a positive association while two studies reported negative associations. Most notably, Inoue et al.’s 2012 study found a positive correlation between several environmental outcomes (i.e., sunlight duration, temperature) and suicide in some prefectures in Japan, while others had significant negative correlations [26]. As the analyses included only a limited adjustment and no tests for interaction, confounding and effect modification could be a possible explanation. Tsai and Cho 2012 reported a positive association between temperature and death by suicide among males and a negative association among females [27]. As many studies did not stratify by gender (Table 5), they would not have been able to observe this relationship.

Studies reporting a significant association were more likely to include a seasonal measure (65.0%) in their analysis as compared to studies reporting no association (33.3%; Table 5). They were also slightly more likely to include confounding variables in their analysis and to stratify their sample by age (15.0% vs. 0.0%) and gender (32.5% vs. 16.7%).

### 3.2. Pollution Factors

The majority of studies assessing the relationship between pollution and suicide included individual-level outcomes (73.9%) rather than population-level outcomes (26.1%). The majority of studies involving an air pollutant reported a significant association between at least one pollutant and death by suicide. More specifically, after stratifying by pollutant type, a slight majority of studies reported a significant association (PM_2.5_: 57.1%; PM_10_: 63.6%; O_3_: 62.5%; NO_2_: 60.0%; and SO_2_: 55.6%; Table 2). Of this stratum, studies commonly reported that NO_2_, O_3_, and PM_10_ had a significant association with suicide. Studies of pesticide (71.4%), toxic metal (100.0%), and noise pollution (100.0%) exposures also tended to report significant associations with death by suicide, although there were fewer studies overall among these groups.

As with the meteorological studies, sample size varied widely regardless of reported results, although those reporting no association had an overall lower median sample size compared to those reporting a significant result (Table 3). It is important to note that sample size ranges were quite large with IQRs upwards of over 100,000 population. Air pollution studies tended to have larger sample sizes as compared to the other pollution types. This may be a result of the number of studies included in this review, particularly because there were fewer studies of pesticide, toxic metal, and noise pollution exposures.

Despite the large proportion of studies reporting a significant result between pollutant and suicide, wide confidence intervals and small effect sizes were common. The majority of studies with a significant result reported a very small or small effect size and all significant results were positive (Table 4). Among the air pollution exposure, only one study reported a moderate effect, while the remaining studies reported very small effects. This study, led by Min (2018), showed a moderate association between changes in IQR of PM_10_ and suicide risk (HR 3.09, 95%CI: 2.63–3.63) [28]. This was a unique finding within the air pollution studies due to its relatively large effect size, although this was one of the only studies reporting hazard ratios. Additionally, concentrations of pollutants were estimated in multiple locations, which may have resulted in over-estimations. All studies of pesticide, toxic metal, and noise pollution exposures reported either a small or very small effect. Among pesticide studies, four out of the five studies reported very small effect sizes. Similarly, both studies on noise pollution reported very small effects.

Assessments of covariates were similar between pollution studies reporting significant and non-significant results (Table 5). Studies with a significant result were slightly more likely to include a measure of season (42.1% vs. 25.0%) and adjust for at least one environmental covariate (52.6% vs. 25.0%). This is largely due to the sheer number of case-crossover studies assessing the relationship between air pollution and death by suicide. A case-crossover design is warranted as it can control for the role of season on pollution, and on air pollution specifically. In particular, a well-powered study by Casas et al. (2017) supported the modifying role of season on the pollution-suicide relationship [29]. They found that seasonality played a role in this association, and as a result, stratified their dataset by season. After stratification, Casas et al. (2017) found a significant association between PM_10_ (OR ranging from 1.02 to 1.07, *p* < 0.05) and suicide only during the summer months. Ozone also had a significant association with suicide, but only during summer, spring and autumn months (OR ranging from 1.02 to 1.07, *p* < 0.05). This observation highlighted the possible modifying role of seasonality, which was not addressed in many other environmental studies in our review. As a result, studies that employed a case-crossover method provided a stronger analysis as they were more comprehensive in accounting for the role of time and season. First, they could control for the role of time and season by carefully choosing control dates within the same week and month as the case date, effectively controlling for month and day effects [29,30,31,32,33,34,35,36,37]. Second, the general time between exposure to a pollutant and death by suicide was a key aspect of several studies. Each case-crossover study addressed the short-term effect of pollution exposure through an analysis of varying lag observations, or the number of days preceding the suicide event. Most commonly, studies addressed lag day 0 through lag day 3. Nine out of the ten case-crossover studies in this exposure group showed at least one significant association between an air pollutant and suicide, and therefore, there is evidence to support the short-term effect of air pollution on death by suicide. Studies of pesticides, noise pollution, and toxic contaminants were less likely to include covariates in their analysis. Notably, Min and Min (2018) found that among younger adults, those exposed to night noise died at a 32% higher rate than that of the control group (HR: 1.32; 95%CI: 1.02–1.70), and hazard rations further increased for older adults and those with at least one mental disorder [38]. There were two notable limitations to this study: (1) the authors had limited noise data on each location and (2) the parent study was not designed for this specific research question, and therefore, the authors did not have access to data on important confounders. As a result, the strength of association is under question. One study addressed suicide risk after exposure to toxic contaminants, including arsenic, lead, mercury, nickel, and uranium [39]. Workers with the highest degree of exposure to toxic metals were more likely to die by suicide, and suicide mortality in those exposed was twice the expected mortality (SMR: 2.1; 95%CI: 1.4–2.7). The results of this study provide a unique early look into the potential effects of toxic metal exposure, although future studies should carefully consider study design and address important covariates and effect modifiers.

### 3.3. Geographic Factors

All geographic exposure studies focused on the role of altitude on suicide risk, and the majority (66.7%) included only population-level outcomes (Table 2). Out of the 12 studies included in this review, eight employed an ecologic or population-level study design and assessed the correlation between city, state, or province altitude and their corresponding suicide rates. Overwhelmingly, these studies found a significant correlation between high altitude and increased suicide rates [40,41,42,43,44,45,46,47,48]. Furthermore, regardless of the type of analysis, studies overwhelmingly reported a significant association between altitude and death by suicide (75.0%) in at least one subpopulation.

Sample size ranged greatly among studies reporting a significant association (Table 3) and interestingly, no studies reporting no association provided a sample size.

Studies of geographic factors did report higher effects as compared to studies of meteorological and pollution factors (Table 4). In fact, four studies (44.4%) reported a moderate effect, a much larger percentage compared to other environmental factors. Furthermore, studies analyzing correlation coefficients reported a small to moderate correlation ranging from Oka, Kubota and Tsubaki (2015)’s study reporting r = 0.462 to Haws et al. (2009)’s study reporting r = 0.74 [43,47]. One study reported a small effect size (r = 0.267), although this study was unique in that it assessed the mean slope for each location and the corresponding standardized mortality ratio. The remaining ecologic studies considered unadjusted and adjusted suicide rates. Selek (2013), a study of Turkish data, was the only ecologic study that did not show a significant correlation between altitude and suicide rate [49]. It is possible that this may be due in part to low numbers of reported suicides because the study included only two years of data (2007–2008). This hypothesis is further supported by a study conducted by Asirdizer (2018) that used the same 81 provinces in Turkey and the same data source [40]. Asirdizer (2018) followed similar methodologies as used by Selek, but included a much large sample size that covered suicide rates for each province for each year between 2006 and 2015. As a result, small numbers of suicides may explain the discrepancy between these two studies. Despite the evidence supporting a correlation between elevation and suicide, there are two notable limitations. First, ecologic fallacy must be considered as a possible explanation. It is possible that although there is a relationship at a population level, these same findings may not be true at an individual level [50]. Second, these studies did not include any individual-level observations, which could have included confounding or modifying variables as described below.

Due to the consistent use of correlation analyses, most studies did not include measures of time or season, or account for confounding or effect modification (Table 5). Confounders and other covariates should be considered in future analyses because those living at high altitude are likely significantly different from those living at low altitude. This is supported by a study by Betz et al. (2011), who found that high and low altitude victims of suicide differ in several key areas: race and ethnicity, rurality, firearm use, and recent financial or personal conflicts [41]. Betz et al. also showed a continued significant difference in mental health status and personal characteristics between low and high-altitude individuals even after multivariate adjustments. A few studies did adjust for confounders, and studies reporting a significant result were more likely to adjust for individual-level (77.8% vs. 0%) and community-level (22.2% vs. 0%) covariates.

Riblet et al. (2019) conducted one of two geographic studies included in this scoping review that included individual-level data [51]. This was a notable cohort study assessing several factors associated with hypoxia, including altitude, smoking, and chronic obstructive pulmonary disease (COPD). After adjusting for age, gender, race and ethnicity, rurality, and VA use, Riblet et al. (2019) reported increasing odds of suicide with each increased level of hypoxic condition. Furthermore, they established a dose–response relationship to these hypoxic factors. Compared to individuals with no hypoxic conditions, those with the highest measured number of conditions (high altitude, current smoker, and diagnosis of COPD) were 3.96 times the odds of death by suicide (95% CI: 3.47, 4.52) after adjustment for demographic variables. Most notably, this study supported the hypothesis of the altitude–suicide relationship rather than simply confirming an association. This study did not adjust for all variables highlighted by Betz et al. (2011), nor was it able to account for severity of COPD or individual use of tobacco, but it provided a basis for future research. Studies addressing these limitations are warranted and supported by the results of this study.

## 4. Discussion

The role of this scoping review was to determine where the literature currently stands on how the environment impacts suicide risk. To date, several reviews have investigated one environmental factor at a time, but no other studies have comprehensively reviewed the role of meteorological, pollution, and geographic variables on death by suicide. The interconnection of these environmental factors is important to consider in suicide risk analysis, both considering their dependent and independent interactions with seasonality. Additionally, these factors can confound or interact each other in certain circumstances. For example, changes in temperature can affect air pollution variables [52]. As a result, considering the importance of these different environmental factors can help to inform future studies on death by suicide.

Overall, we observed that death by suicide is a complex phenomenon not easily summarized at an epidemiologic level. Several factors must be included in predictive modeling or risk analysis, including, but not limited to, individual, community, and environmental factors. Our findings confirm that short-term environmental exposures do impact suicide rates at a population level and suicide risk at an individual level, although environmental factors appear to play only a small role in suicide risk, with effect sizes being small. The strongest factors that should be considered in future research are sunlight exposures, high temperature, air pollution, and home elevation. These risk factors may also be more strongly associated with other measures of suicide behavior, such as suicide attempts. A recent study conducted by Aguglia et al. (2021) reported strong correlations between suicide attempts and temperature, solar radiation, and air pollution (PM2.5) [53]. These results support the significance of temperature, sunlight exposure, and air pollution on suicidal behavior, although the study was limited in that it did not account for individual factors such as psychological or clinical variables. It is possible that while these exposures were only minimally associated with death by suicide, they may play a larger role when considering other measures of suicidal behavior, although this was outside the scope of this review.

### 4.1. Meteorological Factors

Among the meteorological factors, we observed the majority of studies of sunlight intensity, sunlight duration, and temperature reported significant results, although the direction of association varied. This was especially true for sunlight intensity and duration, though there were contradictory results were present in the temperature literature. We have considered two possible explanations. First, there may be specific instances where sunlight or temperature increase or decrease an individual’s risk for suicide, and this may be difficult to quantify at a population level. For example, there may be specific clinical sub-populations of individuals who experience a heightened risk of suicide while other populations experience lowered risk. Studies included in this review often could not account for this possibility due to study design; the majority of meteorological studies were population-based and did not include individual-level covariates that could specifically address the importance of clinical sub-populations. Second, there may be unmeasured confounders (e.g., mental health or medical comorbidities, socio-economic status), which many included studies did not control for. This is a very likely possibility due to the limited adjustment and review of interaction terms across studies. Few studies had access to individual-level covariates to even assess for confounding or effect modification, and as a result, it is very likely that residual confounding existed in most analyses. Given the number of studies and the strength of the study designs, sunlight and high temperature do appear to significantly, if only mildly, affect suicide rates and risk, and should be considered when modeling death by suicide in future studies.

### 4.2. Pollution Factors

Similarly, pollution studies struggled with very small effect sizes, although they clearly reported positive associations when significance was reached. Studies of air pollution, a complex collection of pollutants, consistently reported significant associations with death by suicide after short-term exposures. With the availability of population-level air pollution data, these variables should be assessed as possible covariates when conducting risk assessments in future studies. Pesticide, heavy metal, and noise pollution exposures may also impact suicide risk, though only affect specific populations.

### 4.3. Geographic Factors

Studies assessing a high-altitude exposure provided the highest proportion of small to moderate effect sizes. Despite using data from countries around the world, researchers continued to find significant and moderate associations between high altitude and suicide incidence. This is further supported by the Riblet et al. (2019) cohort study, which included individualized covariates and included analyses to support the hypoxia mechanism hypothesis [51]. It is important to note that individuals living at high altitude differ significantly from those living at low altitude on several measures, including race, ethnicity, and gun ownership [41]. Several studies, due to the population-level study designs, did not account for these differences. Thus, future studies should consider the confounding or modifying role of these covariates in order to more fully investigate how altitude affects suicide risk. Overall, despite limitations to study design, there is evidence to support a small to moderate increase in death by suicide at increasing elevations.

### 4.4. Overview

All studies included in this review shared common strengths and limitations, regardless of environmental factor. This reviews takes into account the innate limitations and strengths of the included studies, and, as a result, the limitations and strengths also refer to limitations and strengths of this review. All studies in this review determined death by suicide based on ICD-9 or ICD-10 codes provided by established agencies. This included using national vital statistics, death records, and medical records. Although local under-reporting may have occurred, this was considered to be a standard accepted outcome measure. Exposure measurements were not as standardized. Exposures were overwhelmingly assigned at a population level because individual-level observations were not available or feasible. They were often measured as daily averages derived from hourly observations at specified locations. The averages were then assigned at a city, county, state, or province level. Although this stabilized measures over time, it introduced the possibility for misclassification bias. Considering that misclassification would likely result in non-differential changes in associations, this would likely bias study results towards to null and could explain the very small effect sizes we observed throughout this review [50]. The second main limitation to studies included in this review was ecologic fallacy. The majority of studies included population-level outcome measures, such as suicide rates. Although significant associations were observed at a population level, it is possible that the associations do not exist at an individual level [50]. It is still unclear as to whether exposure to sunlight or temperature, for example, results in a change in an individual’s suicide risk. Despite these limitations, there is evidence to support further investigation of the role of sunlight, temperature, air pollution, and altitude on suicide risk within a population.

Overall, we identified that several environmental factors do play a significant, though small, role on suicide incidence and risk. It is a small piece to the complex interaction of factors that influence suicide risk at a population level. As a result of the small effect sizes and substantial study design limitations to this field of research, future studies considering suicide as a public health issue should carefully consider whether to include environmental covariates in their analyses. Furthermore, continued research in this field should consider other confounding variables or interaction terms, which may provide more clinically relevant subgroups who experience heightened risk due to any given environmental factor.

### 4.5. Limitations of the Review

This scoping review did not include any analyses to statistically compare studies within each environmental exposure group. Rather, the scoping review methodology allowed us to conduct a standardized review of the current literature and provide overarching observations of studies reporting on the role that the environment may play on suicide risk. The review also included a broad range of study topics, methodologies, and analyses, which are not necessarily comparable. Lastly, observations reported in this review may be affected by publication bias; some studies with non-significant results may not have been published and therefore not included in this review.

## 5. Conclusions

As a result of this review, we aim to inform future studies through a standardized analysis of important covariates, study designs, and current gaps in the literature. As these environmental exposures are potentially modifiable and treatable, it is crucial to gain a more thorough understanding of the environment’s role in suicide risk. As a result of this review, we found that environmental factors are likely of secondary importance to other more substantial factors, such as individual, sociological, or other biological factors. Environmental variables should be considered when modeling or predicting suicide risk as these variables likely confound or interact with other important risk variables. It is unlikely that the environment plays any substantial role on its own in an individual’s suicide risk. Any additional studies considering environmental factors as a primary exposure must include a thorough review of individual-level confounders as this is a gap in the current literature. In summary, the results of this review suggest that several environmental factors (i.e., sunlight intensity, sunlight duration, temperature, air pollution, and altitude) may independently account for only a modest increase in suicide risk. However, certain populations that are at highest risk for suicide may be more sensitive to these additional risks. Further research is warranted in order to account for these additive or multiplicative risks.

## Figures and Tables

**Table 1:** Search terms used for each database.

	Search Terms	Number of Studies
**Medline**		
Meteorology and Suicide	“Sunlight”[Mesh] OR “Ultraviolet Rays”[Mesh] OR “Temperature”[Mesh] OR “Hot Temperature”[Mesh] OR “Cold Temperature”[Mesh] OR “Humidity”[Mesh] OR “Meteorology”[Mesh] OR Sunlight[ti] OR Meterorol*[ti] OR Temperature[ti] or UV[ti] OR Humidity[ti] OR Weather[ti]	202
Pollution and Suicide	“Non-Point Source Pollution”[Mesh] OR “Water Pollution, Radioactive”[Mesh] OR “Water Pollution, Chemical”[Mesh] OR “Water Pollution”[Mesh] OR “Environmental Pollution”[Mesh] OR “Air Pollution, Radioactive”[Mesh] OR “Air Pollution”[Mesh] OR “Traffic-Related Pollution”[Mesh] OR “Noise”[Mesh] OR “Water Pollutants, Chemical”[Mesh] OR “Environmental Pollutants”[Mesh] OR “Vehicle Emissions”[Mesh] OR “Air Pollutants, Radioactive”[Mesh] OR Pollution[ti] OR Pollutants[ti] OR Smog[ti]	339
Geography and Suicide	“Altitude”[Mesh] OR Altitude[ti] or Elevation[ti]	52
**Embase**		
Meteorology and Suicide	Sunlight, Ultraviolet Rays, Temperature, Humidity, Meteorology, or Weather	61
Pollution and Suicide	Pollution, Pollutants, or Smog	26
Geography and Suicide	Altitude or Elevation	25
**PsychInfo**		
Meteorology and Suicide	Sunlight, Ultraviolet, Temperature, Humidity, Meteorology, or Weather	24
Pollution and Suicide	Pollution, Pollutants, or Smog	5
Geography and Suicide	Altitude or Elevation	4

Note: Death by suicide classification is described in the text of the Methods; Acronyms: Mesh, Medical Subject Headings; ti, Title.

**Table 2:** Breakdown of exposures and analysis level of studies included in this review.

		Significant Association *	No Association
Total Studies	Total *N* Studies	*N* (%)	*N* (%)
Meteorological factors			
Primary Exposure			
Sunlight intensity	5	3 (60.0%)	2 (40.0%)
Sunlight duration	23	15 (65.2%)	8 (34.8%)
Temperature	38	30 (78.9%)	6 (15.8%)
Humidity	11	4 (36.4%)	7 (63.6%)
Air pressure	7	3 (42.9%)	4 (57.1%)
Rainfall	16	3 (18.8%)	13 (81.3%)
Primary Analysis			
Population level	34	30 (88.2%)	4 (11.8%)
Individual level	12	10 (83.3%)	2 (16.7%)
Pollution factors			
Primary Exposure			
Air Pollution			
PM_2.5_	7	4 (57.1%)	3 (42.9%)
PM_10_	11	7 (63.6%)	4 (36.4%)
O_3_	8	5 (62.5%)	3 (37.5%)
NO_2_	10	6 (60.0%)	4 (40.0%)
SO_2_	9	5 (55.6%)	4 (44.4%)
Pesticides	7	5 (71.4%)	2 (28.6%)
Toxic Metals	1	1 (100.0%)	0 (0.0%)
Noise	2	2 (100.0%)	0 (0.0%)
Observation Level			
Population	6	4 (66.7%)	2 (33.3%)
Individual	17	15 (88.2%)	2 (11.8%)
Geographic factors ^a^			
Primary Exposure			
Altitude	12	9 (75.0%)	1 (8.3%)
Observation Level			
Population	9	7 (77.8%)	1 (11.1%)
Individual	3	2 (66.7%)	0 (0.0%)

Notes: * Significance at *p* < 0.05. ^a^ Two studies did not report tests of significance. Acronyms: PM_2.5_, particulate matter less than 2.5 micrometers; PM_10_, particulate matter less than 10 micrometers; O_3_, ozone; NO_2_, nitrogen dioxide; SO_2_, sulfur dioxide.

**Table 3:** Descriptive statistics comparing the number of suicides (i.e., sample size) in studies reporting a statistically significant (*p* < 0.05) or non-significant result.

	Significant Association *			No Association		
		Sample Size			Sample Size	
Meteorological Factors	*N* Studies (%) ^b^	Min–Max Number of Suicides	Median (IQR) Number of Suicides	*N* Studies (%) ^b^	Min–Max Number of Suicides	Median (IQR) Number of Suicides
Primary Exposure						
Sunlight intensity	3 (100.0%)	3717–43,393	6600.0 (39,676.0)	1 (50.0%)	3984–3984	3984.0 (0.0)
Sunlight duration	12 (80.0%)	197–128,322	17,378.0 (74,332.5)	6 (75.0%)	536–55,362	6301.0 (6611.0)
Temperature	23 (71.9%)	197–1,320,148	22,564.0 (84,909.0)	3 (50.0%)	3984–7944	5706.0 (3960.0)
Humidity	2 (50.0%)	6600–18,083	12,341.5 (11,483.0)	5 (71.4%)	197–45,293	10,595.0 (21,879.0)
Air pressure	1 (33.3%)	18,083–18,083	18,083.0 (0.0)	2 (50.0%)	685–6600	3642.5 (5915.0)
Rainfall	2 (66.7%)	6600–55,362	30,981.0 (48,762.0)	9 (69.2%)	197–128,322	18,083.0 (37,687.0)
Primary Analysis						
Population level	21 (70.0%)	685–1,320,148	39,347.0 (86,596.0)	1 (25.0%)	3984–3984	3984.0 (0.0)
Individual level	9 (90.0%)	197–69,462	10,595.0 (9569.0)	2 (100.0%)	536–7944	4240.0 (7408.0)
**Pollution Factors**						
Primary Exposure						
Air Pollution						
PM_2.5_	4 (100.0%)	1546–134,811	17,140.0 (79,431.5)	2 (66.7%)	528–1942	1235.0 (1414.0)
PM_10_	6 (85.7%)	564–134,811	12,437.0 (71,895.0)	3 (75.0%)	528–1942	1546.0 (1414.0)
O_3_	4 (80.0%)	1008–73,445	11,267.0 (45,484.5)	2 (66.7%)	528–1942	1235.0 (1414.0)
NO_2_	5 (83.3%)	564–73,445	1550.0 (28,393.0)	3 (75.0%)	528–134,811	1942.0 (134,283.0)
SO_2_	5 (100.0%)	564–134,811	29,939.0 (71,895.0)	2 (50.0%)	1546–1942	1744.0 (396.0)
Pesticides	5 (100.0%)	109–117,469	4991.0 (10,250.0)	2 (100.0%)	90–110	100.0 (20.0)
Toxic Metals	1 (100.0%)	40–40	40.0 (0.0)	n/a	n/a	n/a
Noise	2 (100.0%)	315–528	421.5 (213)	n/a	n/a	n/a
Observation Level						
Population	3 (75.0%)	528–117,469	1008.0 (116,941.0)	1 (50.0%)	1942–1942	1942 (0.0)
Individual	15 (100.0%)	40–134,811	2001.0 (19,969.0)	2 (100.0%)	90–110	100.0 (20.0)
**Geographic Factors ^a^**						
Primary Exposure						
Altitude	3 (33.3%)	22,403–596,704	35,725.0 (574,301.0)	0 (0.0%)	n/a	n/a
Observation Level						
Population	1 (14.3%)	596,704–596,704	596,704 (0.0)	0 (0.0%)	n/a	n/a
Individual	2 (100.0%)	22,403–35,725	29,064.0 (13,322.0)	n/a	n/a	n/a

Notes: * Significance at *p* < 0.05. ^a^ Two studies did not report tests of significance. ^b^ Number of significant/non-significant studies reporting sample size compared to the total number of significant/non-significant studies. Acronyms: IQR, interquartile range; Min, minimum; Max, maximum PM2.5, particulate matter less than 2.5 micrometers; PM_10_, particulate matter less than 10 micrometers; O_3_, ozone; NO_2_, nitrogen dioxide; SO_2_, sulfur dioxide.

**Table 4:** Reported effect sizes among studies reporting a significant result.

	Total *N* Significant Studies *	Very Small Effect	Small Effect	Moderate Effect
Meteorological factors				
Sunlight intensity	3	2 (66.7%)	0 (0.0%)	1 (33.3%)
Sunlight duration	15	6 (40.0%)	6 (40.0%)	3 (20.0%)
Temperature	30	20 (66.7%)	8 (26.7%)	4 (13.3%)
Humidity	4	1 (25.0%)	2 (50.0%)	1 (25.0%)
Air pressure	3	0 (0.0%)	3 (100.0%)	0 (0.0%)
Rainfall	3	2 (66.7%)	1 (33.3%)	0 (0.0%)
Pollution factors				
Air Pollution				
PM_2.5_	4	4 (100.0%)	0 (0.0%)	0 (0.0%)
PM_10_	7	6 (85.7%)	0 (0.0%)	1 (14.3%)
O_3_^c^	5	4 (80.0%)	0 (0.0%)	0 (0.0%)
NO_2_	6	6 (100.0%)	0 (0.0%)	0 (0.0%)
SO_2_	5	5 (100.0%)	0 (0.0%)	0 (0.0%)
Pesticides	5	4 (80.0%)	1 (20.0%)	0 (0.0%)
Toxic Metals	1	0 (0.0%)	1 (100.0%)	0 (0.0%)
Noise	2	2 (100.0%)	0 (0.0%)	0 (0.0%)
Geographic factors ^a,c^				
Altitude	9	2 (22.2%)	2 (22.2%)	4 (44.4%)

Notes: * Significance at *p* < 0.05. ^a^ Two studies did not report statistical significance. ^c^ One study did not report effect size. Acronyms: PM_2.5_, particulate matter less than 2.5 micrometers; PM_10_, particulate matter less than 10 micrometers; O_3_, ozone; NO_2_, nitrogen dioxide; SO_2_, sulfur dioxide.

**Table 5:** Measures of confounding and effect modification across studies included in review.

				Adjustment for Confounding			Effect Modification		
	Total *N* Studies	Measure of Time ^d^	Measure of Season ^e^	Environment Covariates	Individual-Level Factors	Community-Level Factors	Age	Gender	Suicide Method
Meteorological factors									
Significant association *	40	14 (35.0%)	26 (65.0%)	8 (20.0%)	3 (7.5%)	3 (7.5%)	6 (15.0%)	13 (32.5%)	3 (7.5%)
No association	6	2 (33.3%)	2 (33.3%)	1 (16.7%)	0 (0.0%)	0 (0.0%)	0 (0.0%)	1 (16.7%)	1 (16.7%)
Pollution factors									
Significant association *	19	10 (52.6%)	8 (42.1%)	10 (52.6%)	7 (36.8%)	0 (0.0%)	9 (47.4%)	11 (57.9%)	3 (15.8%)
No association	4	3 (75.0%)	1 (25.0%)	1 (25.0%)	2 (50.0%)	0 (0.0%)	2 (50.0%)	2 (50.0%)	0 (0.0%)
Geographic factors ^a^									
Significant association *	9	0 (0.0%)	0 (0.0%)	0 (0.0%)	7 (77.8%)	2 (22.2%)	0 (0.0%)	0 (0.0%)	0 (0.0%)
No association	1	0 (0.0%)	0 (0.0%)	0 (0.0%)	0 (0.0%)	0 (0.0%)	0 (0.0%)	0 (0.0%)	0 (0.0%)

Notes: * Significance at *p* < 0.05. ^a^ Two studies did not report statistical significance. ^d^ Measures of time included studies addressing the time between exposure and outcome (e.g., lag). ^e^ Measures of season included adjustment, stratification, or matching by season or month variables.

## Data Availability

The authors did not create any new data for this study; rather, information was abstracted directly from studies included in this review (listed in Word S1). Data sharing is not applicable in this article.

## Supplementary Materials

The following are available online at https://www.mdpi.com/article/10.3390/ijerph18157809/s1, Word S1: Citations of studies included in this scoping review.
