# Supplementary material for: The Effect of Meteorological, Pollution, and Geographic Exposures on Death by Suicide: A Scoping Review
Source: Int J Environ Res Public Health. 2021 Jul 23;18(15):7809. doi: 10.3390/ijerph18157809 (PMC8345465; doi:10.3390/ijerph18157809)
Supplement: Supplementary file 1 [file ijerph-18-07809-s001.zip › ijerph-1275952-supplementary.pdf]

Citations of Studies Included in This Scoping Review

Ajdacic-Gross, V., Lauber, C., Sansossio, R., Bopp, M., Eich, D., Gostynski, M., Rossler, W. (2007). Seasonal associations between weather conditions and suicide—Evidence against a classic hypothesis. American Journal of Epidemiology, 165(5), 561–569.

Asirdizer, M., Kartal, E., Etli, Y., Tatlisumak, E., Gumus, O., Hekimoglu, Y., & Keskin, S. (2018). The effect of altitude and climate on the suicide rates in Turkey. J Forensic Leg Med, 54, 91–95.

Astudillo-García, C. I., Rodríguez-Villamizar, L. A., Cortez-Lugo, M., Cruz-De la Cruz, J. C., & Fernández-Niño, J. A. (2019). Air Pollution and Suicide in Mexico City: A Time Series Analysis, 2000–2016. Int J Environ Res Public Health, 16(16), 2971.

Bakian, A. V., Huber, R. S., Coon, H., Gray, D., Wilson, P., McMahon, W. M., & Renshaw, P. F. (2015). Acute air pollution exposure and risk of suicide completion. Am J Epidemiol, 181(5), 295–303.

Bando, D. H., Teng, C. T., Volpe, F. M., Masi, E., Pereira, L. A., & Braga, A. L. (2017). Suicide and meteorological factors in São Paulo, Brazil, 1996–2011: a time series analysis. Braz J Psychiatry, 39(3), 220–227.

Beard, J. D., Umbach, D. M., Hoppin, J. A., Richards, M., Alavanja, M. C., Blair, A., Kamel, F. (2011). Suicide and pesticide use among pesticide applicators and their spouses in the agricultural health study. Environ Health Perspect, 119(11), 1610–1615.

Betz, M. E., Valley, M. A., Lowenstein, S. R., Hedegaard, H., Thomas, D., Stallones, L., & Honigman, B. (2011). Elevated suicide rates at high altitude: sociodemographic and health issues may be to blame. Suicide Life Threat Behav, 41(5), 562–573.

Biermann, T., Stilianakis, N., Bleich, S., Thürauf, N., Kornhuber, J., & Reulbach, U. (2009). The hypothesis of an impact of ozone on the occurrence of completed and attempted suicides. Med Hypotheses, 72(3), 338–341.

Bozsonyi, K., Lester, D., Fulop, A., Zonda, T., & Balint, L. (2020). The effects of sunshine duration and ambient temperature on suicides in Hungary. Neuropsychopharmacol Hung, 22(1), 23–28.

Brenner, B., Cheng, D., Clark, S., & Camargo, C. A., Jr. (2011). Positive association between altitude and suicide in 2584 U.S. counties. High Alt Med Biol, 12(1), 31–35.

Carleton, T. A. (2017). Crop-damaging temperatures increase suicide rates in India. Proc Natl Acad Sci U S A, 114(33), 8746–8751.

Casas, L., Cox, B., Bauwelinck, M., Nemery, B., Deboosere, P., & Nawrot, T. S. (2017). Does air pollution trigger suicide? A case-crossover analysis of suicide deaths over the life span. Eur J Epidemiol, 32(11), 973–981.

Chau, P. H., Yip, P. S. F., Lau, H. Y. E., Ip, Y. T., Law, F. Y. W., Ho, R. T. H., Woo, J. (2020). Hot Weather and Suicide Deaths among Older Adults in Hong Kong, 1976–2014: A Retrospective Study. Int J Environ Res Public Health, 17(10).

Díaz, J., López-Bueno, J. A., López-Ossorio, J. J., Gónzález, J. L., Sánchez, F., & Linares, C. (2020). Short-term effects of traffic noise on suicides and emergency hospital admissions due to anxiety and depression in Madrid (Spain). Sci Total Environ, 710, 136315.

Dixon, P. G., & Kalkstein, A. J. (2018). Where are weather-suicide associations valid? An examination of nine US counties with varying seasonality. Int J Biometeorol, 62(5), 685–697.

Dixon, P. G., McDonald, A. N., Scheitlin, K. N., Stapleton, J. E., Allen, J. S., Carter, W. M., Roberts, J. B. (2007). Effects of temperature variation on suicide in five U.S. counties, 1991–2001. Int J Biometeorol, 51(5), 395–403.

Dixon, P. G., Sinyor, M., Schaffer, A., Levitt, A., Haney, C. R., Ellis, K. N., & Sheridan, S. C. (2014). Association of weekly suicide rates with temperature anomalies in two different climate types. Int J Environ Res Public Health, 11(11), 11627–11644.

Faria, N. M., Fassa, A. G., & Meucci, R. D. (2014). Association between pesticide exposure and suicide rates in Brazil. Neurotoxicology, 45, 355–362.

Fernández-Arteaga, V., Tovilla-Zárate, C. A., Fresán, A., González-Castro, T. B., Juárez-Rojop, I. E., López-Narváez, L., & Hernández-Díaz, Y. (2016). Association between completed suicide and environmental temperature in a Mexican population, using the Knowledge Discovery in Database approach. Comput Methods Programs Biomed, 135, 219–224.

Fernández-Niño, J. A., Astudillo-García, C. I., Rodríguez-Villamizar, L. A., & Florez-Garcia, V. A. (2018). Association between air pollution and suicide: a time series analysis in four Colombian cities. Environ Health, 17(1), 47.

Fernández-Niño, J. A., Flórez-García, V. A., Astudillo-García, C. I., & Rodríguez-Villamizar, L. A. (2018). Weather and Suicide: A Decade Analysis in the Five Largest Capital Cities of Colombia. Int J Environ Res Public Health, 15(7), 1313.

Figgs, L. W., Holsinger, H., Freitas, S. J., Brion, G. M., Hornung, R. W., Rice, C. H., & Tollerud, D. (2011). Increased suicide risk among workers following toxic metal exposure at the Paducah gaseous diffusion plant from 1952 to 2003: a cohort study. Int J Occup Environ Med, 2(4), 199–214.

Grjibovski, A. M., Kozhakhmetova, G., Kosbayeva, A., & Menne, B. (2013). Associations between air temperature and daily suicide counts in Astana, Kazakhstan. Medicina (Kaunas), 49(8), 379–385.

Ha, H., & Tu, W. (2018). An Ecological Study on the Spatially Varying Relationship between County-Level Suicide Rates and Altitude in the United States. Int J Environ Res Public Health, 15(4), 671.

Haws, C. A., Gray, D. D., Yurgelun-Todd, D. A., Moskos, M., Meyer, L. J., & Renshaw, P. F. (2009). The possible effect of altitude on regional variation in suicide rates. Med Hypotheses, 73(4), 587–590.

Helama, S., Holopainen, J., & Partonen, T. (2013). Temperature-associated suicide mortality: contrasting roles of climatic warming and the suicide prevention program in Finland. Environ Health Prev Med, 18(5), 349–355.

Hernández, O. H., Hernández-Sánchez, J. A., & Flores-Gutiérrez, J. D. (2018). Annual fluctuations of sunlight and suicides in a region South of the Tropic of Cancer. Biological Rhythm Research, 49(3), 405–411. Retrieved from http://search.ebscohost.com/login.aspx?direct=true&AuthType=ip,athens&db=psyh&AN=2018–15420-005&site=ehost-live ORCID: 0000–0002-7269–5716 ohhernan@uacam.mx

Hiltunen, L., Haukka, J., Ruuhela, R., Suominen, K., & Partonen, T. (2014). Local daily temperatures, thermal seasons, and suicide rates in Finland from 1974 to 2010. Environ Health Prev Med, 19(4), 286–294.

Holopainen, J., Helama, S., Björkenstam, C., & Partonen, T. (2013). Variation and seasonal patterns of suicide mortality in Finland and Sweden since the 1750s. Environ Health Prev Med, 18(6), 494–501.

Holopainen, J., Helama, S., & Partonen, T. (2014). Does diurnal temperature range influence seasonal suicide mortality? Assessment of daily data of the Helsinki metropolitan area from 1973 to 2010. Int J Biometeorol, 58(6), 1039–1045.

Huber, R. S., Coon, H., Kim, N., Renshaw, P. F., & Kondo, D. G. (2014). Altitude is a risk factor for completed suicide in bipolar disorder. Med Hypotheses, 82(3), 377–381.

Inoue, K., Nishimura, Y., Fujita, Y., Ono, Y., & Fukunaga, T. (2012). The relationship between suicide and five climate issues in a large-scale and long-term study in Japan. West Indian Med J, 61(5), 532–537.

Kim, C., Jung, S. H., Kang, D. R., Kim, H. C., Moon, K. T., Hur, N. W., Suh, I. (2010). Ambient particulate matter as a risk factor for suicide. Am J Psychiatry, 167(9), 1100–1107.

Kim, J., Choi, N., Lee, Y. J., An, H., Kim, N., Yoon, H. K., & Lee, H. J. (2014). High altitude remains associated with elevated suicide rates after adjusting for socioeconomic status: a study from South Korea. Psychiatry Investig, 11(4), 492–494.

Kim, N., Mickelson, J. B., Brenner, B. E., Haws, C. A., Yurgelun-Todd, D. A., & Renshaw, P. F. (2011). Altitude, gun ownership, rural areas, and suicide. Am J Psychiatry, 168(1), 49–54.

Kim, Y., Kim, H., Gasparrini, A., Armstrong, B., Honda, Y., Chung, Y., Hashizume, M. (2019). Suicide and Ambient Temperature: A Multi-Country Multi-City Study. Environ Health Perspect, 127(11), 117007.

Kim, Y., Kim, H., Honda, Y., Guo, Y. L., Chen, B. Y., Woo, J. M., & Ebi, K. L. (2016). Suicide and Ambient Temperature in East Asian Countries: A Time-Stratified Case-Crossover Analysis. Environ Health Perspect, 124(1), 75–80.

Kim, Y., Kim, H., & Kim, D.-S. (2011). Association between daily environmental temperature and suicide mortality in Korea (2001–2005). Psychiatry Research, 186(2), 390–396.

Kim, Y., Myung, W., Won, H. H., Shim, S., Jeon, H. J., Choi, J., Kim, D. K. (2015). Association between air pollution and suicide in South Korea: a nationwide study. PLoS One, 10(2), e0117929.

Kim, Y., Ng, C. F. S., Chung, Y., Kim, H., Honda, Y., Guo, Y. L., Hashizume, M. (2018). Air Pollution and Suicide in 10 Cities in Northeast Asia: A Time-Stratified Case-Crossover Analysis. Environ Health Perspect, 126(3), 037002.

Lambert, G., Reid, C., Kaye, D., Jennings, G., & Esler, M. (2003). Increased suicide rate in the middle-aged and its association with hours of sunlight. Am J Psychiatry, 160(4), 793–795.

Lee, H., Myung, W., Kim, S. E., Kim, D. K., & Kim, H. (2018). Ambient air pollution and completed suicide in 26 South Korean cities: Effect modification by demographic and socioeconomic factors. Sci Total Environ, 639, 944–951.

Lee, H. C., Lin, H. C., Tsai, S. Y., Li, C. Y., Chen, C. C., & Huang, C. C. (2006). Suicide rates and the association with climate: a population-based study. J Affect Disord, 92(2), 221–226.

Lester, D. (1986). Suicide and homicide rates: their relationship to latitude and longitude and to the weather. Suicide Life Threat Behav, 16(3), 356–359.

Lin, G. Z., Li, L., Song, Y. F., Zhou, Y. X., Shen, S. Q., & Ou, C. Q. (2016). The impact of ambient air pollution on suicide mortality: a case-crossover study in Guangzhou, China. Environ Health, 15(1), 90.

Likhvar, V., Honda, Y., & Ono, M. (2011). Relation between temperature and suicide mortality in Japan in the presence of other confounding factors using time-series analysis with a semiparametric approach. Environ Health Prev Med, 16(1), 36–43.

Luan, G., Yin, P., Wang, L., & Zhou, M. (2019). Associations between ambient high temperatures and suicide mortality: a multi-city time-series study in China. Environ Sci Pollut Res Int, 26(20), 20377–20385.

MacFarlane, E., Simpson, P., Benke, G., & Sim, M. R. (2011). Suicide in Australian pesticide-exposed workers. Occup Med (Lond), 61(4), 259–264.

Maes, M., De Meyer, F., Thompson, P., Peeters, D., & Cosyns, P. (1994). Synchronized annual rhythms in violent suicide rate, ambient temperature and the light-dark span. Acta Psychiatr Scand, 90(5), 391–396.

Makris, G. D., Reutfors, J., Larsson, R., Isacsson, G., Ösby, U., Ekbom, A., Papadopoulos, F. C. (2016). Serotonergic medication enhances the association between suicide and sunshine. J Affect Disord, 189, 276–281.

Marion, S. A., Agbayewa, M. O., & Wiggins, S. (1999). The effect of season and weather on suicide rates in the elderly in British Columbia. Can J Public Health, 90(6), 418–422.

Meyer, A., Koifman, S., Koifman, R. J., Moreira, J. C., de Rezende Chrisman, J., & Abreu-Villaca, Y. (2010). Mood disorders hospitalizations, suicide attempts, and suicide mortality among agricultural workers and residents in an area with intensive use of pesticides in Brazil. J Toxicol Environ Health A, 73(13), 866–877.

Min, J. Y., Kim, H. J., & Min, K. B. (2018). Long-term exposure to air pollution and the risk of suicide death: A population-based cohort study. Sci Total Environ, 628–629, 573–579.

Min, J. Y., & Min, K. B. (2018). Night noise exposure and risk of death by suicide in adults living in metropolitan areas. Depress Anxiety, 35(9), 876–883.

Nejar, K. A., Benseñor, I. M., & Lotufo, P. A. (2007). Sunshine and suicide at the tropic of Capricorn, São Paulo, Brazil, 1996-2004. Rev Saude Publica, 41(6), 1062-1064.

Ng, C. F., Stickley, A., Konishi, S., & Watanabe, C. (2016). Ambient air pollution and suicide in Tokyo, 2001–2011. J Affect Disord, 201, 194–202.

Oka, M., Kubota, T., Tsubaki, H., & Yamauchi, K. (2015). Analysis of impact of geographic characteristics on suicide rate and visualization of result with Geographic Information System. Psychiatry Clin Neurosci, 69(6), 375–382.

Page, L. A., Hajat, S., & Kovats, R. S. (2007). Relationship between daily suicide counts and temperature in England and Wales. Br J Psychiatry, 191, 106–112.

Papadopoulos, F. C., Frangakis, C. E., Skalkidou, A., Petridou, E., Stevens, R. G., & Trichopoulos, D. (2005). Exploring lag and duration effect of sunshine in triggering suicide. J Affect Disord, 88(3), 287–297.

Petridou, E., Papadopoulos, F. C., Frangakis, C. E., Skalkidou, A., & Trichopoulos, D. (2002). A role of sunshine in the triggering of suicide. Epidemiology, 13(1), 106–109.

Pickett, W., King, W. D., Lees, R. E., Bienefeld, M., Morrison, H. I., & Brison, R. J. (1998). Suicide mortality and pesticide use among Canadian farmers. Am J Ind Med, 34(4), 364–372.

Preti, A. (1998). The influence of climate on suicidal behaviour in Italy. Psychiatry Res, 78(1), 9–19.

Qi, X., Hu, W., Mengersen, K., & Tong, S. (2014). Socio-environmental drivers and suicide in Australia: Bayesian spatial analysis. BMC Public Health, 14, 681.

Riblet, N. B., Gottlieb, D. J., Watts, B. V., Cornelius, S. L., Fan, V. S., Shi, X., & Shiner, B. (2019). Hypoxia-related risk factors for death by suicide in a national clinical sample. Psychiatry Res, 273, 247–251.

Ruuhela, R., Hiltunen, L., Venäläinen, A., Pirinen, P., & Partonen, T. (2009). Climate impact on suicide rates in Finland from 1971 to 2003. Int J Biometeorol, 53(2), 167–175.

Sabic, H., Kious, B., Boxer, D., Fitzgerald, C., Riley, C., Scholl, L., Kondo, D. G. (2019). Effect of Altitude on Veteran Suicide Rates. High Alt Med Biol, 20(2), 171–177.

Salib, E., & Gray, N. (1997). Weather conditions and fatal self-harm in North Cheshire 1989-1993. Br J Psychiatry, 171, 473-477.

Santurtún, M., Sanchez-Lorenzo, A., Del Real, Á., Zarrabeitia, M. T., & Santurtún, A. (2018). Association Between Suicide and Environmental Variables in the North of Spain: A 14-Year Analysis. Cult Med Psychiatry, 42(3), 647–653.

Schneider, A., Hampel, R., Ladwig, K. H., Baumert, J., Lukaschek, K., Peters, A., & Breitner, S. (2020). Impact of meteorological parameters on suicide mortality rates: A case-crossover analysis in Southern Germany (1990–2006). Sci Total Environ, 707, 136053.

Selek, S. (2013). Altitude, immigration and suicide rates: a study from Turkey. Psychiatry Investig, 10(1), 89–91.

Seregi, B., Kapitány, B., Maróti-Agóts, Á., Rihmer, Z., Gonda, X., & Döme, P. (2017). Weak associations between the daily number of suicide cases and amount of daily sunlight. Prog Neuropsychopharmacol Biol Psychiatry, 73, 41–48.

Sim, K., Kim, Y., Hashizume, M., Gasparrini, A., Armstrong, B., Sera, F., Chung, Y. (2020). Nonlinear temperature-suicide association in Japan from 1972 to 2015: Its heterogeneity and the role of climate, demographic, and socioeconomic factors. Environ Int, 142, 105829.

Stallones, L. (2006). Suicide and potential occupational exposure to pesticides, Colorado 1990–1999. J Agromedicine, 11(3), 107–112.

Tietjen, G. H., & Kripke, D. F. (1994). Suicides in California (1968–1977): absence of seasonality in Los Angeles and Sacramento counties. Psychiatry Res, 53(2), 161–172.

Tsai, J. F., & Cho, W. (2012). Temperature change dominates the suicidal seasonality in Taiwan: a time-series analysis. J Affect Disord, 136(3), 412–418.

van Wijngaarden, E. (2003). An exploratory investigation of suicide and occupational exposure. J Occup Environ Med, 45(1), 96–101.

van Wijngaarden, E., & Savitz, D. A. (2000). Occupational sunlight exposure in relation to suicide among electric utility workers. Am J Ind Med, 38(2), 149–154.

Vyssoki, B., Kapusta, N. D., Praschak-Rieder, N., Dorffner, G., & Willeit, M. (2014). Direct effect of sunshine on suicide. JAMA Psychiatry, 71(11), 1231–1237.

Vyssoki, B., Praschak-Rieder, N., Sonneck, G., Blüml, V., Willeit, M., Kasper, S., & Kapusta, N. D. (2012). Effects of sunshine on suicide rates. Compr Psychiatry, 53(5), 535–539.

Yan, Y. Y. (2000). Geophysical variables and behavior: LXXXXIX. The influence of weather on suicide in Hong Kong. Percept Mot Skills, 91(2), 571–577.

Yang, C. Y., Weng, Y. H., & Chiu, Y. W. (2019). Relationship between ozone air pollution and daily suicide mortality: a time-stratified case-crossover study in Taipei. J Toxicol Environ Health A, 82(4), 261–267.
